# Plant‐Derived Polyphenol Salvianolic Acid B Attenuates Cardiomyocyte Pyroptosis by Modulating the MMP12–NLRP3 Signaling Axis

**DOI:** 10.1002/fsn3.72300

**Published:** 2026-09-30

**Authors:** Zilong Xie, Zhi‐Kai Yang, Longhui Zheng, Pinghua Wu, Shengxian Chen, Xiaotong Ye, Yi Gao

**Affiliations:** ^1^ Department of Cardiovascular Medicine Fujian Medical University Union Hospital Fuzhou China; ^2^ Department of General Medicine Xiamen Chang Gung Hospital, Huaqiao University Xiamen China; ^3^ School of Medicine Xiamen University Xiamen China; ^4^ School of Medicine Huaqiao University Quanzhou China

**Keywords:** hyperglycemia‐induced cardiomyocyte injury, matrix metalloproteinase 12, metabolic cardiomyopathy, NLRP3 inflammasome, pyroptosis, salvianolic acid B

## Abstract

Plant‐derived polyphenols are widely investigated as bioactive compounds with potential relevance to cardiometabolic health. Salvianolic acid B (Sal B), a major phenolic compound from *Salvia miltiorrhiza* , has been reported to exhibit antioxidant and anti‐inflammatory activities; however, its role in cardiomyocyte pyroptosis remains uncertain. In this study, a high glucose (HG)‐stimulated AC16 cardiomyocyte injury model was established to recapitulate the hyperglycemic injury phenotype observed in metabolic cardiomyopathy. Combined functional assays, bioinformatic mining, molecular docking, and matrix metalloproteinase 12 (MMP12) overexpression rescue experiments were performed to explore the protective effects and underlying molecular mechanisms of Sal B activity. The results revealed that Sal B mitigated HG‐triggered oxidative stress and inflammatory response and repressed nucleotide‐binding domain, leucine‐rich–containing family, pyrin domain–containing‐3 (NLRP3)‐dependent cardiomyocyte pyroptosis. MMP12 was identified as a key molecular mediator associated with the protective effects of Sal B, and MMP12 overexpression abrogated the cytoprotective effects of Sal B. These findings provide mechanistic evidence that Sal B modulates MMP12–NLRP3‐associated pyroptotic signaling in cardiomyocyte injury models, supporting further investigation of plant‐derived polyphenols in cardiometabolic nutrition research.

## Graphical abstract

HG induces MMP12 upregulation in AC16 cardiomyocytes, which activates the NLRP3 inflammasome, triggers GSDMD‐mediated pyroptosis, and promotes the release of inflammatory cytokines. Sal B alleviates cardiomyocyte damage by modulating MMP12, while MMP12 overexpression abrogates this protective effect. GSDMD, gasdermin D; HG, high glucose; MMP12, matrix metalloproteinase 12; NLRP3, nucleotide‐binding domain, leucine‐rich–containing family, pyrin domain–containing‐3; Sal B, salvianolic acid B.

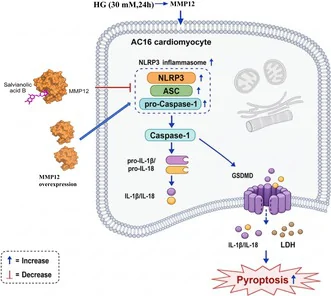

## 1 Introduction

*Salvia miltiorrhiza* is an edible plant with medicinal properties that is renowned for its ability to promote blood circulation and eliminate blood stasis (Wei et al. 2023), and salvianolic acid B (Sal B) is a well‐characterized natural small‐molecule monomer derived from this plant (Figure 1A) (Zhao et al. 2024). Sal B can activate the Nrf2 signaling pathway, enhance the endogenous antioxidant defense system, effectively scavenge reactive oxygen species (ROS), and alleviate oxidative stress damage (Ji et al. 2020; Wang et al. 2025). Sal B exerts marked cytoprotective effects against myocardial ischemia/reperfusion injury by inhibiting ferroptosis and regulating apoptosis and autophagy (Xu et al. 2023; Zhang et al. 2020). Additionally, Sal B exhibits immunomodulatory functions, influencing macrophage polarization and immune cell migration, and plays a regulatory role in tissue repair and inflammation (Hu, Yang, et al. 2024; Qin et al. 2024). These findings establish Sal B as a multifunctional plant‐derived bioactive compound with antioxidant, anti‐inflammatory, and tissue‐protective bioactivities.

**FIGURE 1:**
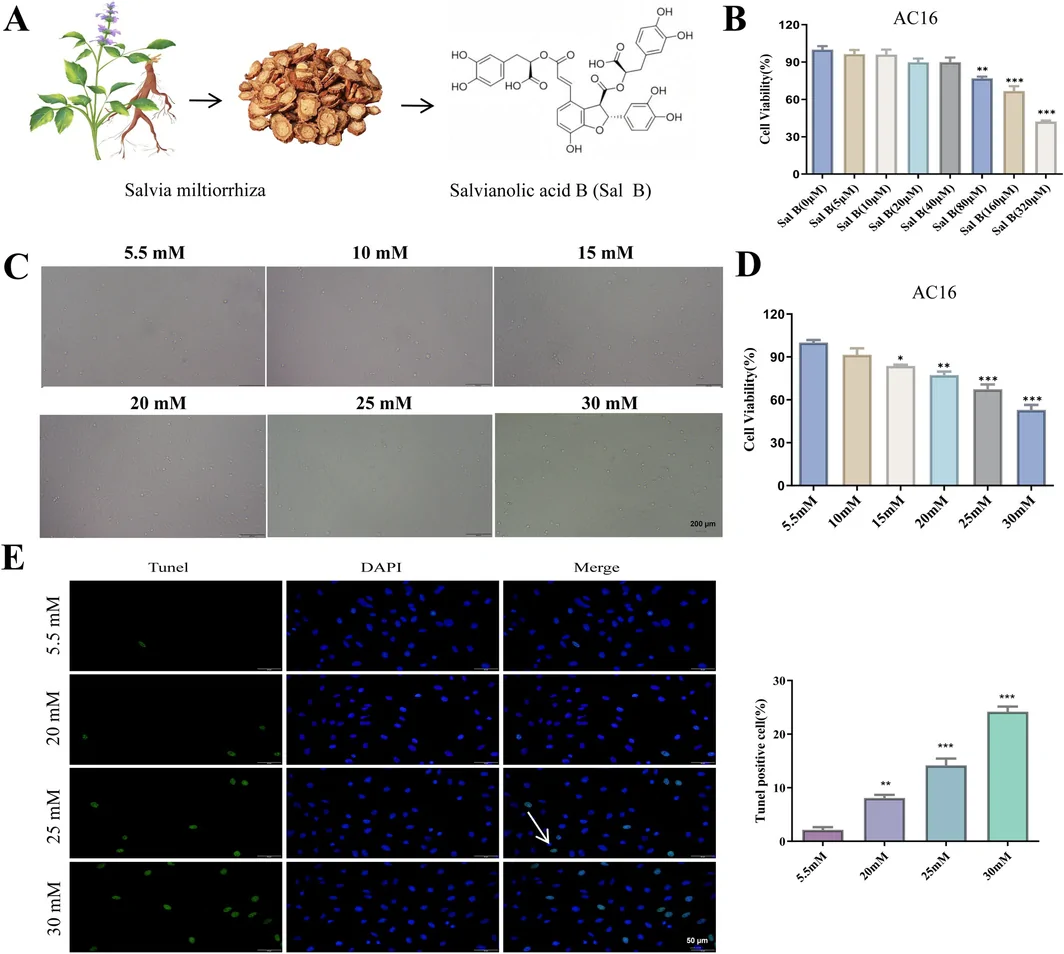
Establishment of HG‐induced cardiomyocyte injury model and cytotoxicity assessment of Sal B (A) Natural source and chemical structure of Sal B (B) Determination of the optimal concentration of Sal B in AC16 cardiomyocytes (C–E) Establishment of HG‐induced injury model in AC16 cardiomyocytes; arrow indicates a representative TUNEL‐positive cell. Data are shown as mean ± standard deviation. (*p < 0.05, **p < 0.01, ***p < 0.001 vs. control group. *n* = 3). DAPI, 4′,6‐diamidino‐2‐phenylindole; HG, high glucose; Sal B, salvianolic acid B.

Metabolic cardiomyopathy (MCM) is an important cardiovascular complication associated with metabolic diseases such as diabetes and obesity, significantly increasing the risk of heart failure and malignant arrhythmias (Costantino et al. 2023; Donath et al. 2019). Its core pathological mechanism involves imbalanced myocardial energy metabolism, lipotoxic injury, and chronic inflammation‐mediated myocardial remodeling (Fossier et al. 2022; Ren et al. 2021; Shang et al. 2024; Yuan et al. 2024). In the field of cardiometabolic nutrition, nutritional strategies based on cellular protective mechanisms that target the core pathological processes of MCM and have favorable safety profiles are urgently needed. Although the beneficial cardiometabolic bioactivities of Sal B have been suggested, the specific mechanisms underlying its effects in MCM, particularly its modulation of key inflammatory injury pathways, remain insufficiently characterized.

Epidemiological and experimental studies have linked dietary polyphenol intake with reduced risk of cardiometabolic disorders (Mendonça et al. 2019), partially through alleviating intracellular oxidative stress and chronic low‐grade inflammation (Dama et al. 2024; Deng et al. 2026). As a water‐soluble polyphenol from *S. miltiorrhiza* , Sal B has been widely studied in the context of functional foods and nutritional supplements (Younis et al. 2026). However, the current understanding of its effects on metabolic cardiomyocyte injury is mostly limited to conventional apoptosis and oxidative stress pathways. Only a few studies have addressed its regulatory role in pyroptotic signaling from a nutritional mechanistic perspective. Elucidating the cytoprotective mechanisms of Sal B at the cellular level can deepen our understanding of the nutritional bioactivities of edible plant‐derived polyphenols and provide preliminary mechanistic evidence for developing cardiometabolic health‐oriented functional food ingredients and targeted nutritional intervention strategies. In this context, recent studies have shown that pyroptosis triggered by activation of the nucleotide‐binding domain, leucine‐rich–containing family, pyrin domain–containing‐3 (NLRP3) inflammasome (Bai et al. 2025) is a key link connecting metabolic disorders and myocardial inflammatory injury (Sharma and Kanneganti 2021; Toldo and Abbate 2024). Metabolic stress factors such as hyperglycemia can lead to excessive activation of the NLRP3 inflammasome in cardiomyocytes, resulting in gasdermin D (GSDMD)‐mediated membrane pore formation and the massive release of proinflammatory factors such as interleukin (IL)‐1β and IL‐18, directly exacerbating myocardial injury and dysfunction (Sun and Ding 2021; Vande Walle and Lamkanfi 2024). However, the upstream mechanisms driving the abnormal activation of NLRP3 inflammasome in cardiomyocytes under metabolic stress require further elucidation.

Matrix metalloproteinase 12 (MMP12) is significantly upregulated in atherosclerotic cardiovascular diseases. Based on the known biochemical characteristics and pathological functions of MMP12, we hypothesized that MMP12 promotes NLRP3 inflammasome activation through two nonmutually exclusive pathways. First, MMP12 facilitates the priming phase of NLRP3 inflammasome activation via damage‐associated molecular pattern (DAMP) production. MMP12 is known to preferentially cleave extracellular matrix (ECM) components including elastin and fibronectin (Luo et al. 2026). The ECM serves as a reservoir for high mobility group box 1 (HMGB1); MMP12‐driven ECM degradation consequently liberates sequestered HMGB1 and generates ECM‐derived DAMP fragments. These DAMPs, encompassing extracellular HMGB1, engage pattern recognition receptors to initiate nuclear factor‐κB signaling and upregulate transcription of *NLRP3* and proinflammatory cytokines, thereby establishing the priming signal for NLRP3 inflammasome activation (Wang et al. 2021). Second, MMP12 may contribute to the activation signal required for NLRP3 inflammasome assembly via a positive inflammatory feedback loop. Accumulating evidence indicates that MMP12 enhances macrophage recruitment and infiltration into metabolically injured tissues (Nighot et al. 2021). Tissue‐infiltrating macrophages amplify local inflammatory cascades and propagate tissue metabolic stress, which further drives the generation of secondary danger signals to trigger NLRP3 inflammasome oligomerization. Accordingly, we proposed that MMP12‐mediated macrophage infiltration and subsequent inflammatory amplification provide the second signal enabling full NLRP3 inflammasome activation. Consistently, in vivo MMP12 knockout alleviates metabolic disorders and suppresses tissue inflammation (Amor et al. 2026), supporting MMP12 as an upstream modulator of NLRP3 signaling and a critical mediator in metabolic cardiovascular injury. Although Sal B exerts potent anti‐inflammatory and cardioprotective properties, whether Sal B ameliorates hyperglycemia‐induced cardiomyocyte injury by suppressing hyperglycemia‐triggered cardiomyocyte pyroptosis through the modulation of MMP12 remains poorly defined in the context of hyperglycemic MCM.

Therefore, we aimed to verify the cytoprotective effect of Sal B against high glucose (HG)‐induced cardiomyocyte injury and investigate the role of the MMP12–NLRP3–pyroptosis signaling axis in this process. These findings provide preliminary mechanistic evidence linking Sal B to the modulation of pyroptotic signaling for future research on plant‐derived polyphenol nutritional strategies targeting cardiometabolic health.

## 2 Materials and Methods

### 2.1 Reagents

The AC16 human cardiomyocyte cell line was provided by the School of Medicine, Xiamen University, China. Sal B (SKU: BD17651) was procured from Shanghai Bide Pharmaceutical Technology Co. Ltd. (China). Fetal bovine serum (SKU: SH30084.03) was acquired from HyClone (USA). Phosphate‐buffered saline (PBS; SKU: C10010500BT) was sourced from Life Technologies (USA), and trypsin (SKU: 25200072) was obtained from Gibco (USA). The Cell Counting Kit‐8 (CCK‐8) Assay Kit (SKU: CK04) was provided by Sinochem Research Institute (China). The Single‐Step TUNEL Apoptosis Detection Kit (SKU: C1088) was provided by Shanghai Biocytogen Biosciences Co. Ltd. (Shanghai, China). The Reactive Oxygen Species Assay Kit (SKU: CA1410) was provided by Beijing Solarbio Biotech Co. Ltd. (China). Wuhan Elabscience (China) supplied the Human tumor necrosis factor (TNF)‐α enzyme‐linked immunosorbent assay (ELISA) Kit (SKU: E‐EL‐H0109), Human IL‐18 ELISA Kit (SKU: E‐EL‐H0253), and Human IL‐1β ELISA Kit (SKU: E‐EL‐H0149). The RNAprep FastPure Tissue & Cell Kit (SKU: TSP413), SynScriptIII RT SuperMix for quantitative polymerase chain reaction (qPCR) (+gDNA Remover) (SKU: TSK314M), and ArtiCanA SYBR qPCR Mix (SKU: TSE501) were purchased from Beijing Qingke Biotechnology Co. Ltd. (China). The following antibodies were used: anti‐NLRP3 (SKU: 19771‐1‐AP, 1:500, Proteintech, China; and SKU: DF7438, 1:100, Affinity Biosciences, China), anti‐β‐actin (SKU: 20536‐1‐AP, 1:5000, Proteintech), anti‐apoptosis‐associated speck‐like protein containing a CARD (ASC; SKU: bs‐6741R, 1:500, Bioss, China), anti‐caspase‐1 (SKU: 3866T, 1:1000, Cell Signaling Technology, USA; and SKU: 81482‐1‐RR, 1:100, Proteintech), anti‐GSDMD (SKU: 1:500, Affinity Biosciences), horseradish peroxidase (HRP)‐conjugated goat anti‐rabbit IgG (SKU: 111‐035‐003, 1:10,000, Jackson Immunoresearch, USA), HRP‐conjugated goat anti‐mouse IgG (SKU: 115‐035‐003, 1:10,000, Jackson Immunoresearch), and Andy Fluor 488 Goat Anti‐Rabbit IgG (H + L) Antibody (SKU: L110B, 1:200, ABP Biosciences, China). All other reagents were of domestic analytical grade.

### 2.2 Instruments

The microplate reader (Multiskan MK3) was provided by Thermo Scientific (USA); the refrigerated centrifuge (Neofuge‐15R), CO_2_ incubator (HF90), and biological safety cabinet (HF‐1200LC) were supplied by Shanghai Heal Force (China). The inverted microscope (CKX53) was supplied by Olympus (Japan), and the low‐speed centrifuge (5702R) was supplied by Eppendorf (Germany). The cell counter (Cellometer Mini) was provided by Nexcelom (Germany); the analytical flow cytometer (CytoFLEX) was supplied by Beckman Coulter (USA), and the horizontal shaker (TS‐1) was provided by Haimen Qilinbeier Instrument Manufacturing Co. Ltd. (China). The benchtop high‐speed refrigerated centrifuge (HT190R) was provided by Hunan Xiangyi Laboratory Instrument Development Co. Ltd. (China); the real‐time qPCR system (ViiA 7) was supplied by ABI (USA). The ultra‐micro spectrophotometer (K5600) was provided by Beijing Zokmoon Technology Co. Ltd. (China), and the horizontal electrophoresis system (JY300) was supplied by Beijing Junyi Dongfang Electrophoresis Equipment Co. Ltd. (China).

### 2.3 Establishment of HG‐Induced AC16 Cardiomyocyte Injury Model

AC16 cardiomyocytes were treated with a series of glucose concentrations to establish an in vitro injury model mimicking MCM. Referring to widely used protocols in cellular models of diabetic cardiomyopathy, 5.5 mmol/L glucose served as the normal physiological control, while 30 mmol/L glucose was selected for the HG injury group. AC16 cardiomyocytes in the logarithmic growth phase were cultured at 37°C with 95% air and 5% CO_2_ for 24 h. Glucose solutions were prepared at concentrations of 5.5, 10, 15, 20, 25, and 30 mmol/L and mixed by vortexing. The cell culture medium was then discarded, and 100 μL of each glucose concentration was added to 96‐well plates and cultured under 5% CO_2_ and 37°C conditions for an additional 24 h.

### 2.4 Determination of Optimal Concentration of Sal B

The concentration range of Sal B was determined based on published in vitro studies on cardiomyocytes, and the final working concentration was determined based on cytotoxicity assessments to ensure cell viability above 90%. Sal B at concentrations of 0, 10, 20, 40, 80, 160, and 320 μmol/L was added to the culture medium. Cells were inoculated into 96‐well plates (100 μL/well) and incubated overnight at 37°C under 5% CO_2_. Subsequently, 10 μL of CCK‐8 solution was added to each well, and the plates were incubated at 37°C under 5% CO_2_ for 3 h. Absorbance values were measured at 450 nm to determine cell viability across the groups and the optimal noncytotoxic working concentration.

### 2.5 Determination of Lactate Dehydrogenase, Superoxide Dismutase, and Malondialdehyde Activities

After treatment of each group of cells, the cell culture supernatants were collected, and levels of lactate dehydrogenase (LDH), superoxide dismutase (SOD), and malondialdehyde (MDA) were measured at 450 nm with the microplate reader according to the manufacturer's instructions.

### 2.6 TUNEL Assay

Cells were seeded into 24‐well plates at a density of 3.5 × 10^4^ cells/well. After 24 h of treatment with glucose solutions (5.5–30 mmol/L), the cells were fixed with 4% paraformaldehyde for 30 min, permeabilized with 0.3% Triton X‐100 for 5 min, and incubated with TUNEL detection buffer (50 μL/well) at 37°C in the dark for 60 min. After washing with PBS, the slides were sealed with an anti‐fluorescence quenching medium and observed under a fluorescence microscope (excitation 450–500 nm/emission 515–565 nm) at 200× and 400× magnifications. Under uniform imaging parameters, five random fields per coverslip were acquired at 200× magnification. Two investigators performed blinded, independent cell counting with cross‐validated results.

### 2.7 ELISAs for IL‐1β, IL‐18, and TNF‐α

Cell supernatants from each group were collected, and the levels of inflammatory mediators (IL‐1β, IL‐18, and TNF‐α) were measured according to the manufacturer's instructions.

### 2.8 Flow Cytometry

Cells were centrifuged at 150 *g* for 5 min to collect the pellet. The pellet was resuspended in serum‐free medium containing 10 μmol/L 2′,7′‐dichlorodihydrofluorescein diacetate and incubated at 37°C under 5% CO_2_ in a humidified atmosphere for 20 min, with intermittent vortexing every 5 min. Following incubation, the cells were centrifuged at 150 *g* for 5 min, and the supernatant was discarded. The cells were subsequently washed three times with prechilled serum‐free medium. Finally, the cells were resuspended in prechilled PBS, and the fluorescence intensity was immediately quantified using flow cytometry (fluorescein isothiocyanate channel) to determine intracellular ROS levels.

### 2.9 Immunofluorescence Staining

AC16 cardiomyocytes in logarithmic growth phase were seeded onto sterile glass coverslips in 24‐well plates at a density of 3.5 × 10^4^ cells per well, cultured for 24 h, and pretreated with 40 μmol/L Sal B for 24 h followed by 30 mmol/L HG stimulation for another 24 h. After rinsing with PBS, the cells were fixed with 4% paraformaldehyde for 30 min at room temperature, permeabilized with 0.5% Triton X‐100 for 20 min, and blocked with 5% normal goat serum for 30 min. The coverslips were then incubated with primary antibodies against NLRP3 and caspase‐1 overnight at 4°C, washed thoroughly, and incubated with species‐matched fluorescence‐conjugated secondary antibodies for 60 min at room temperature in the dark. Nuclei were counterstained with 4′,6‐diamidino‐2‐phenylindole for 5 min, and the coverslips were mounted with anti‐fluorescence quenching mounting medium; fluorescent images were acquired at 200× and 400× magnifications under a fluorescence microscope. Six random fields per coverslip were imaged at 400× magnification with consistent settings. Blinded, independent semi‐quantification was conducted by two researchers, with cross‐validated outcomes.

### 2.10 Reverse Transcription qPCR

Total RNA was extracted using a Cell Total RNA Extraction Kit, followed by reverse transcription to synthesize cDNA. The qPCR analysis was conducted according to the 2× TSINGKE Master qPCR Mix protocol, with the reaction mixture containing 2 μL reverse transcription product, 10 μL SYBR Green Mix, 0.8 μL forward and reverse primers, and 6 μL sterile water. The reaction conditions were 95°C for 10 s, 60°C for 60 s, and 72°C for 15 s, with a total of 40 cycles. Real‐time fluorescence signals were collected during the reaction, and *ACTB* was used as a stable endogenous reference gene for normalization. Gene expression changes were analyzed using the 2^−ΔΔCt^ method, and specific primer sequences are listed in Table 1.

**TABLE 1:** Primer sequences of genes.

Gene name		Sequence (5′‐3′)	Size
*NLRP3*	Forward	GTTTGACCCCGATGATGAGC	244 bp
	Reverse	CTTGTGGATGGGTGGGTTTG	
*CASP1*	Forward	GCTGAGGTTGACATCACAGGCA	145 bp
	Reverse	TGCTGTCAGAGGTCTTGTGCTC	
*ASC*	Forward	GCCGAGGAGCTCAAGAAGTT	116 bp
	Reverse	TTGTCGGTGAGGTCCAAGG	
*GSDMD*	Forward	GCTTCCACTTCTACGATGCC	163 bp
	Reverse	AGAGTCTGCCAGGTGTTAGG	
*β‐actin*	Forward	CATGTACGTTGCTATCCAGGC	250 bp
	Reverse	CTCCTTAATGTCACGCACGAT	
*MMP12*	Forward	GATCTGGCATTGGAGGGGAT	193 bp
	Reverse	GCAGAGAGGCGAAATGTGTT	

### 2.11 Western Blot

Total protein was extracted from AC16 cells using a T‐PER Protein Extraction Kit. After quantification, the extracted proteins underwent electrophoresis and were transferred to a polyvinylidene fluoride (PVDF) membrane. The membrane was blocked with 5% skim milk/Tris‐buffered saline with Tween‐20 at room temperature for 1 h and incubated overnight at 4°C with primary antibody, followed by secondary antibody incubation at room temperature for 2 h. The enhanced chemiluminescence reagents were diluted in ddH_2_O, mixed thoroughly before use, and used to incubate the PVDF membrane for signal development; the results were observed using a gel imaging system. The gray values of protein bands were determined, and the relative expression levels of the proteins of interest were calculated using β‐actin as an internal reference.

### 2.12 Analysis of Gene Expression Data

Key regulatory factors were identified using multidimensional analysis of GSE205891 (a human clinical dataset consisting of muscle tissue samples from patients with diabetes and healthy controls) and GSE76811 (a dataset consisting of gene expression profiles from white adipose tissue and aortae of obese and atherosclerotic mice; this dataset does not include cardiac tissue but has been previously used to successfully identify MMP12 as a key regulator in metabolic inflammation) from the Gene Expression Omnibus (GEO) database. Differential expression analysis was performed using the limma software package (version 3.50.0) in the R programming language. The significance thresholds were set at |log_2_FC| > 1 and adjusted *p*‐value < 0.05 after Benjamini–Hochberg correction. Weighted gene co‐expression network analysis (WGCNA, R package version 1.70‐3) was used to construct a weighted gene co‐expression network, with a soft threshold power of *β* = 9 (*R* ^2^ = 0.85) to establish a scale‐free topology (Xu et al. 2022). MCM‐related gene sets were downloaded from the UniProt database using the keyword “metabolic cardiomyopathy”. The Venn intersection of differentially expressed genes, turquoise module genes (most strongly correlated with MCM phenotype), and UniProt MCM gene set was determined to screen candidate genes. The classification performance of MMP12 was evaluated using a receiver operating characteristic (ROC) curve.

### 2.13 Molecular Docking

The crystal structure of human MMP12 (PDB ID: 3LIL, resolution 1.8 Å) was retrieved from the RCSB Protein Data Bank. The three‐dimensional structure of Sal B (PubChem CID: 6451084) was downloaded from the PubChem database in structure data format and converted to MOL2 format for subsequent processing. Protein preparation was performed as follows: crystallographic water molecules were removed from the MMP12 structure, polar hydrogen atoms were added, and nonpolar hydrogens were merged; atomic charges were assigned using the Gasteiger method under the AutoDock force field. The optimized structure was used as the protein receptor for docking analysis. Initial rapid conformation screening was performed using the LibDock module in Discovery Studio to preliminarily evaluate the binding orientation of Sal B in the MMP12 active pocket. For accurate binding affinity calculations and conformation optimization, semi‐flexible molecular docking was further conducted using AutoDock Vina 1.2.5. Before docking, both ligand and receptor files were converted to PDBQT format via AutoDock Tools 1.5.7, with Gasteiger charges assigned to the ligand. The grid box was centered on the catalytic Zn^2+^ ion in the MMP12 active site, with dimensions of 20 Å × 20 Å × 20 Å to fully cover the entire binding cavity. The exhaustiveness parameter was set to 8, and nine independent binding poses were generated per docking run. The pose with the lowest binding free energy was selected as the optimal docking conformation for the subsequent interaction analysis. Binding patterns including hydrogen bonds, hydrophobic interactions, and π–π stacking were visualized and comprehensively analyzed using PyMOL 2.5 (Gaillard 2018).

### 2.14 Construction of MMP12 Overexpression Plasmid

The coding sequence of MMP12 was retrieved from the National Center for Biotechnology Information. Primers containing EcoRI/XhoI restriction enzyme cleavage sites were designed to synthesize the full‐length MMP12 coding sequence. The double‐digested MMP12 fragment was ligated to the pcDNA3.1(+) vector using T4 DNA ligase at 16°C overnight. Subsequently, the ligation products were transformed into competent DH5α *Escherichia coli* and spread on ampicillin‐supplemented Luria–Bertani plates, followed by incubation at 37°C for 16 h. Single clones were selected, and plasmids were extracted after preliminary screening using colony PCR. The sequences were verified using double‐enzyme digestion and Sanger sequencing to ensure accuracy.

### 2.15 Data and Statistical Analysis

All quantitative data are presented as the mean ± standard deviation from at least three independent experiments. Statistical analyses were performed using GraphPad Prism 9.5. Data distribution was assessed before hypothesis testing. For multi‐group comparisons, one‐way analysis of variance followed by Dunnett's post hoc tests was used for normally distributed data with equal variance, while the Kruskal–Wallis nonparametric test was used for non‐normally distributed data. For two‐group comparisons, Student's unpaired *t*‐test or Welch's *t*‐test was used.

## 3 Results

### 3.1 Establishment of HG‐Induced Cardiomyocyte Injury Model and Cytotoxicity Assessment of Sal B

After treatment with Sal B at concentrations of 0, 10, 20, 40, 80, 160, and 320 μmol/L for 24 h, the non‐cytotoxic working concentration for AC16 cardiomyocytes was determined to be 40 μmol/L (Figure 1B). Treatment of AC16 cells with glucose at concentrations of 5.5–30 mmol/L for 24 h produced a concentration‐dependent increase in cell death, with the most pronounced effect observed at 30 mmol/L, as confirmed by light microscopy, CCK‐8 assay, and TUNEL staining (Figure 1C–E).

### 3.2 Sal B Attenuates HG‐Induced Injury in AC16 Cardiomyocytes

To investigate the protective effects of Sal B against HG‐induced cardiomyocyte injury, AC16 cells were pretreated with 40 μmol/L Sal B before HG exposure, with the normal‐glucose group serving as the control. HG stimulation significantly reduced cell viability compared with the control treatment (*p* < 0.001), whereas Sal B pretreatment markedly improved cell viability (*p* < 0.001 vs. HG group) (Figure 2A). ELISA results further showed that Sal B significantly suppressed HG‐induced release of proinflammatory cytokines, including IL‐1β, IL‐18, and TNF‐α (Figure 2B). In addition, Sal B attenuated HG‐induced intracellular ROS accumulation (Figure 2C). Consistently, Sal B treatment significantly reversed HG‐induced increases in MDA and LDH levels, together with the decrease in SOD activity (Figure 2D), suggesting an improvement in oxidative stress status and cellular injury. Furthermore, TUNEL staining demonstrated that Sal B reduced HG‐induced cell death (Figure 2E). Collectively, these findings indicate that Sal B protects against HG‐induced cardiomyocyte injury by alleviating oxidative stress and inflammatory damage.

**FIGURE 2:**
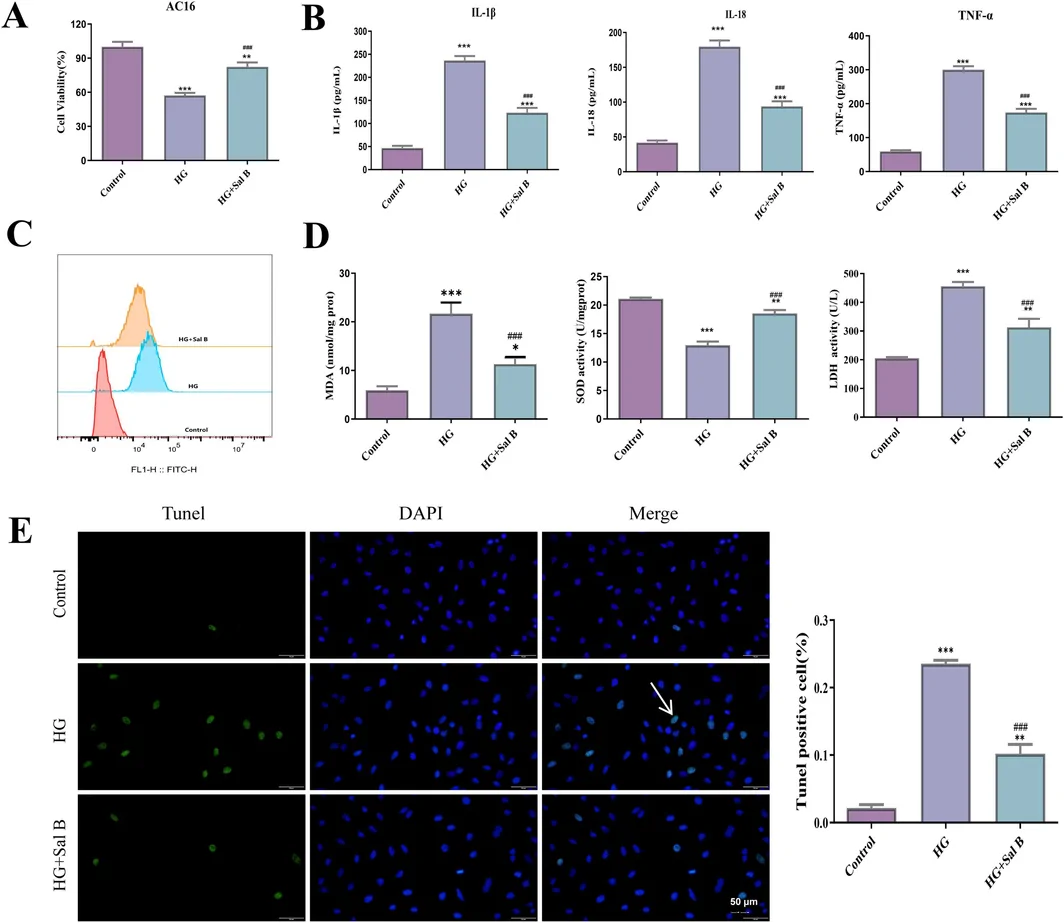
Sal B attenuates HG‐induced injury in AC16 cardiomyocytes. (A) Cell Counting Kit‐8 assay assessing cell viability. (B) Enzyme‐linked immunosorbent assay quantification of proinflammatory cytokines IL‐1β, IL‐18, and TNF‐α in cell culture supernatants. (C) Flow cytometric measurement of intracellular reactive oxygen species. (D) Biochemical quantification of oxidative stress markers MDA and SOD, and the cytotoxicity marker LDH. (E) TUNEL fluorescence staining for quantifying DNA fragmentation as an indicator of cell death in AC16 cardiomyocytes; arrow indicates a representative TUNEL‐positive cell. Data are shown as mean ± standard deviation. (*p < 0.05, **p < 0.01, ***p < 0.001 vs. control group; ^###^ *p* < 0.001 vs. HG group. *n* = 3). DAPI, 4′,6‐diamidino‐2‐phenylindole; HG, high glucose; IL, interleukin; LDH, lactate dehydrogenase; MDA, malondialdehyde; Sal B, salvianolic acid B; SOD, superoxide dismutase; TNF, tumor necrosis factor.

### 3.3 Sal B Attenuates HG‐Induced Cardiomyocyte Injury by Modulating NLRP3 Inflammasome‐Associated Pyroptotic Signaling

To investigate the effect of Sal B on pyroptotic signaling under HG conditions, we detected key molecules of the NLRP3 inflammasome pathway and pyroptosis executors, combined with cell death and inflammatory cytokine assays. The cells were divided into three groups: Control, HG, and HG + Sal B. Immunofluorescence analysis showed that HG stimulation markedly increased the protein expression of NLRP3 and caspase‐1 compared with control treatment (*p* < 0.001; Figure 3A), whereas Sal B treatment significantly attenuated these increases. Consistent with these observations, reverse transcription qPCR (RT‐qPCR) analysis demonstrated that the mRNA levels of *NLRP3*, *ASC*, *CASP1*, and *GSDMD* were significantly elevated after HG stimulation but were reduced after Sal B treatment (Figure 3B). Western blot analysis further confirmed that HG exposure increased the protein expression of NLRP3, ASC, cleaved caspase‐1, and the active N‐terminal fragment of GSDMD (GSDMD‐N), whereas Sal B pretreatment partially reversed these changes (Figure 3C). These results suggest that Sal B suppresses HG‐induced activation of the NLRP3 inflammasome‐related pyroptosis pathway in cardiomyocytes.

**FIGURE 3:**
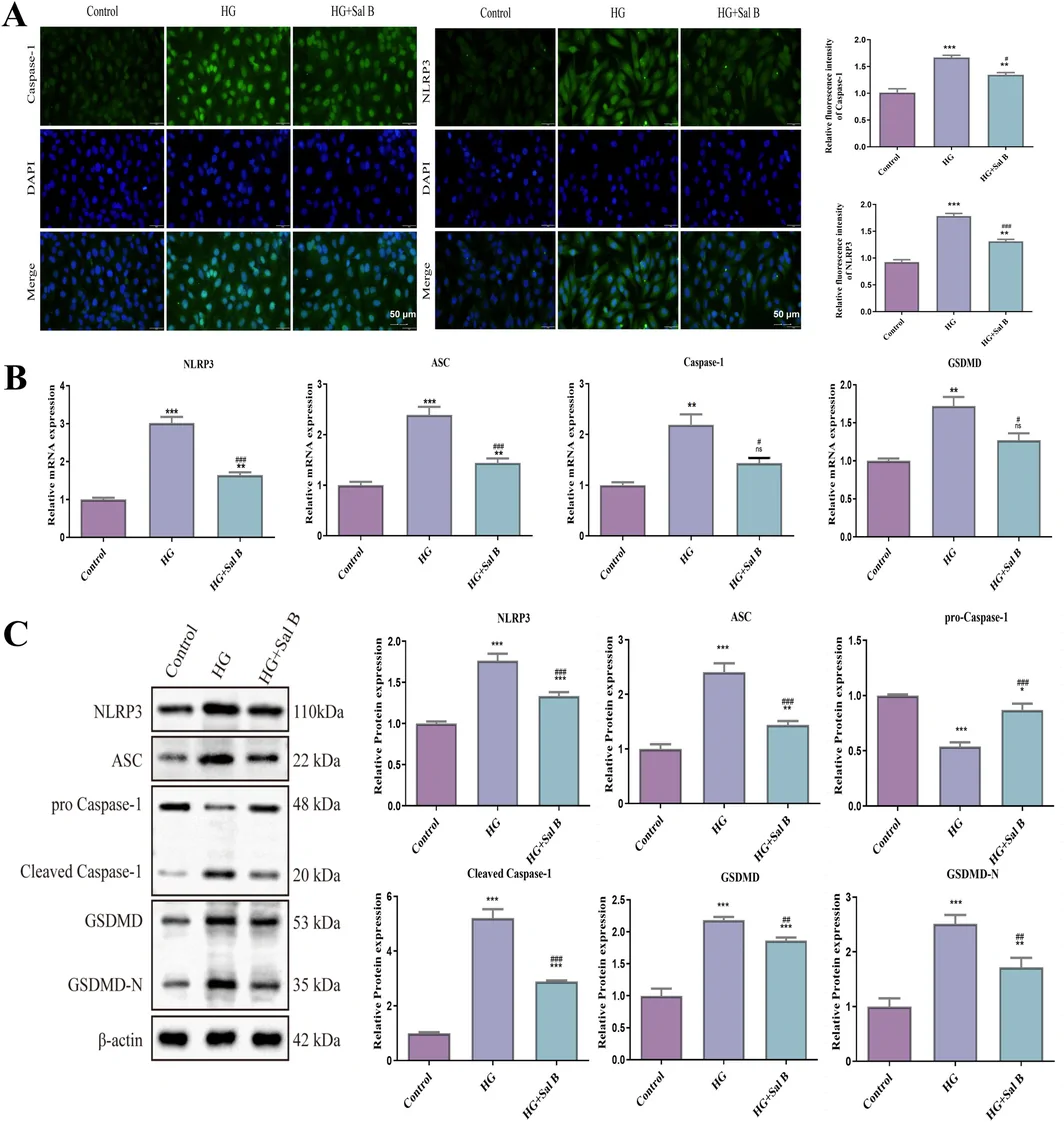
Sal B attenuates HG‐induced cardiomyocyte injury via modulating NLRP3 inflammasome‐associated pyroptotic signaling. (A) Immunofluorescence detection of caspase‐1 and NLRP3. (B) Reverse transcription quantitative polymerase chain reaction profiling of mRNA expressions: *NLRP3*, *ASC*, *CASP1* (*Caspase‐1*), and *GSDMD*. (C) Protein expressions via western blot: NLRP3, ASC, pro‐caspase‐1, cleaved‐caspase‐1, GSDMD, and GSDMD‐N. Data are shown as mean ± standard deviation. (*p < 0.05, **p < 0.01 ***p < 0.001 vs. control group; ^#^ *p* < 0.05, ^##^ *p* < 0.01, ^###^ *p* < 0.001 vs. HG group. *n* = 3). ASC, apoptosis‐associated speck‐like protein containing a CARD; GSDMD, gasdermin D; GSDMD‐N, active N‐terminal fragment of GSDMD; HG, high glucose; NLRP3, nucleotide‐binding domain, leucine‐rich–containing family, pyrin domain–containing‐3; ns, not significant; Sal B, salvianolic acid B.

### 3.4 Bioinformatic Identification of MMP12 as a Candidate Molecular Mediator Associated With MCM

To explore potential molecular mediators involved in MCM, we analyzed publicly available transcriptomic datasets from the GEO database, including the human clinical dataset GSE205891 and animal model dataset GSE76811. First, differential expression analysis was performed on both datasets using the limma algorithm, with significance thresholds set at |log_2_FC| > 1 and adjusted *p* < 0.05 (Benjamini–Hochberg correction), to identify genes aberrantly expressed under MCM conditions. Subsequently, WGCNA was performed on the GSE205891 dataset to construct a scale‐free gene coexpression network and partition genes into functional modules. The turquoise module, which exhibited the strongest correlation with MCM phenotypic traits (cor = 0.99, *p* < 1e‐200), was selected as the core module closely associated with disease progression.

To narrow down candidate genes, we conducted Venn intersection analysis among three gene sets: the differentially expressed genes from the two datasets, turquoise module hub genes from WGCNA, and MCM‐related genes annotated in the UniProt database. As a result, *MMP12* was identified as the only overlapping candidate gene associated with MCM (Figure 4A–H). Preliminary evaluation via ROC curve analysis showed that *MMP12* exhibited favorable discriminatory performance for MCM status in the discovery dataset (Figure 4I). Notably, this result was derived from a relatively small sample cohort and was only used as a reference for preliminary candidate screening; its actual diagnostic value should be verified in larger independent cohorts. Further Spearman correlation analysis confirmed that *MMP12* expression was positively correlated with multiple disease‐related molecular signatures, including inflammatory response and ECM remodeling gene sets, further supporting its functional involvement in MCM pathological processes (Figure 4J).

**FIGURE 4:**
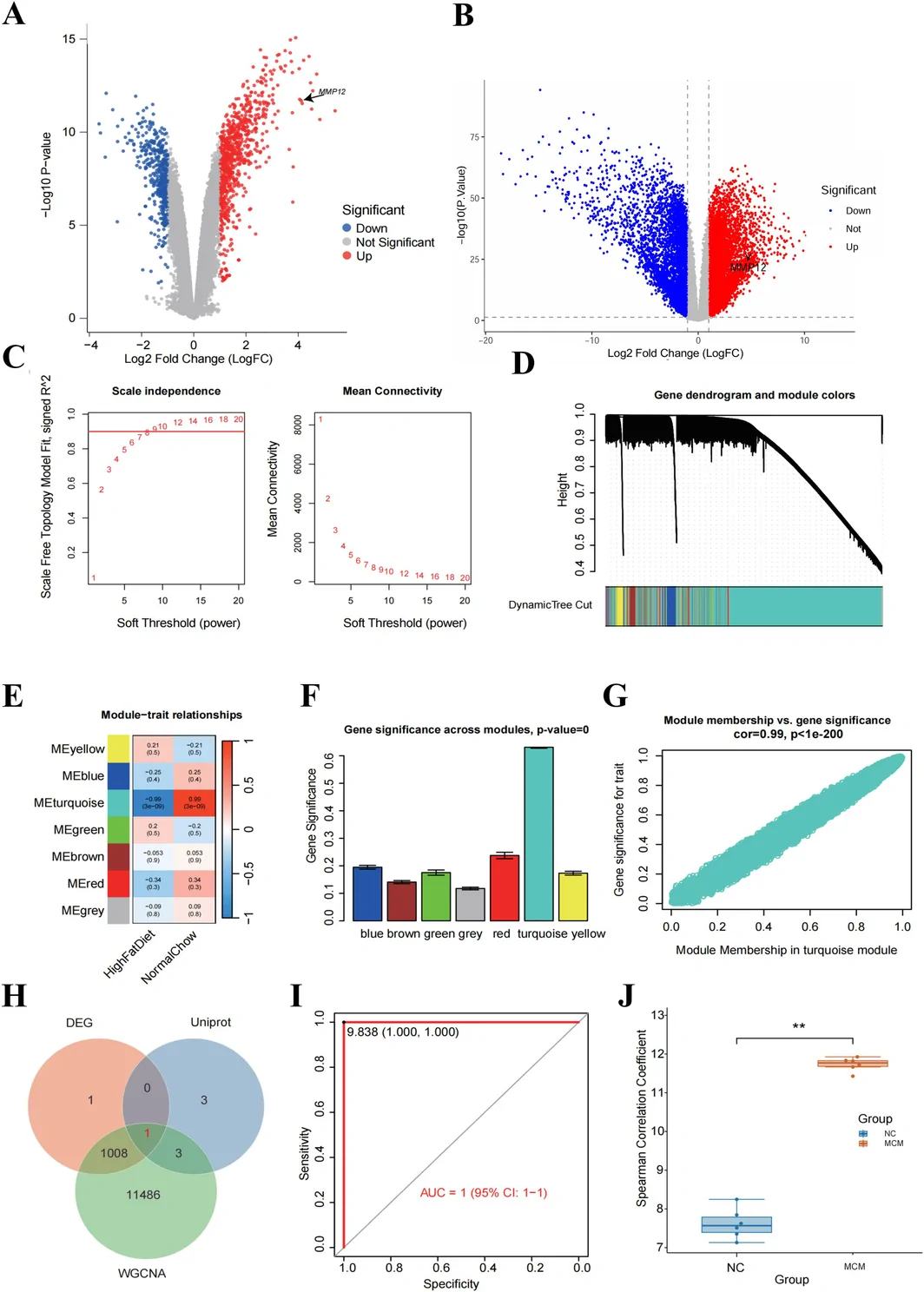
Bioinformatic identification of MMP12 as a candidate molecular mediator associated with MCM. (A) Limma volcano plot of DEGs in the GSE205891 dataset. (B) Limma volcano plot of DEGs in the GSE76811 dataset. (C) Weighted gene co‐expression network analysis. (D) Dynamic clustering dendrogram. (E) Relationship between consensus modules and samples. (F) Cross‐module gene significance. (G) Turquoise module representing MCM gene significance. (H) Venn diagram showing intersection of DEGs, turquoise‐colored module genes, and UniProt gene set identifying one candidate gene. (I) ROC curve evaluating the discriminatory performance of MMP12 in the discovery dataset. (J) Spearman correlation coefficient plot. AUC, area under the curve; DEGs, differentially expressed genes; MCM, metabolic cardiomyopathy; MMP12, matrix metalloproteinase 12; ROC, receiver operating characteristic.

### 3.5 Molecular Docking Analysis of Sal B With MMP12

The docking results showed that Sal B could stably dock into the active cavity of MMP12, with a minimum binding free energy of −9.2 kcal/mol. This value is markedly lower than the conventional threshold (−5.0 kcal/mol) for favorable biomolecular recognition, indicating a strong theoretical binding affinity between Sal B and MMP12. Further structural dissection revealed that the stable binding was jointly maintained by multiple noncovalent intermolecular forces. Specifically, Sal B formed hydrogen bond interactions with the key residues Lys233 and Thr239 in the binding pocket, which anchored the compound in the active cavity. Hydrophobic interactions with residues Phe114 and Leu117 further enhanced the binding stability. Additionally, a π–π stacking interaction was formed between Sal B and Tyr242, a conserved residue located in the S1′ specificity pocket of MMP12. Collectively, these intermolecular forces may facilitate the stable occupancy of Sal B in the functional pocket of MMP12, thereby potentially modulating its proteolytic activity (Figure 5A–D).

**FIGURE 5:**
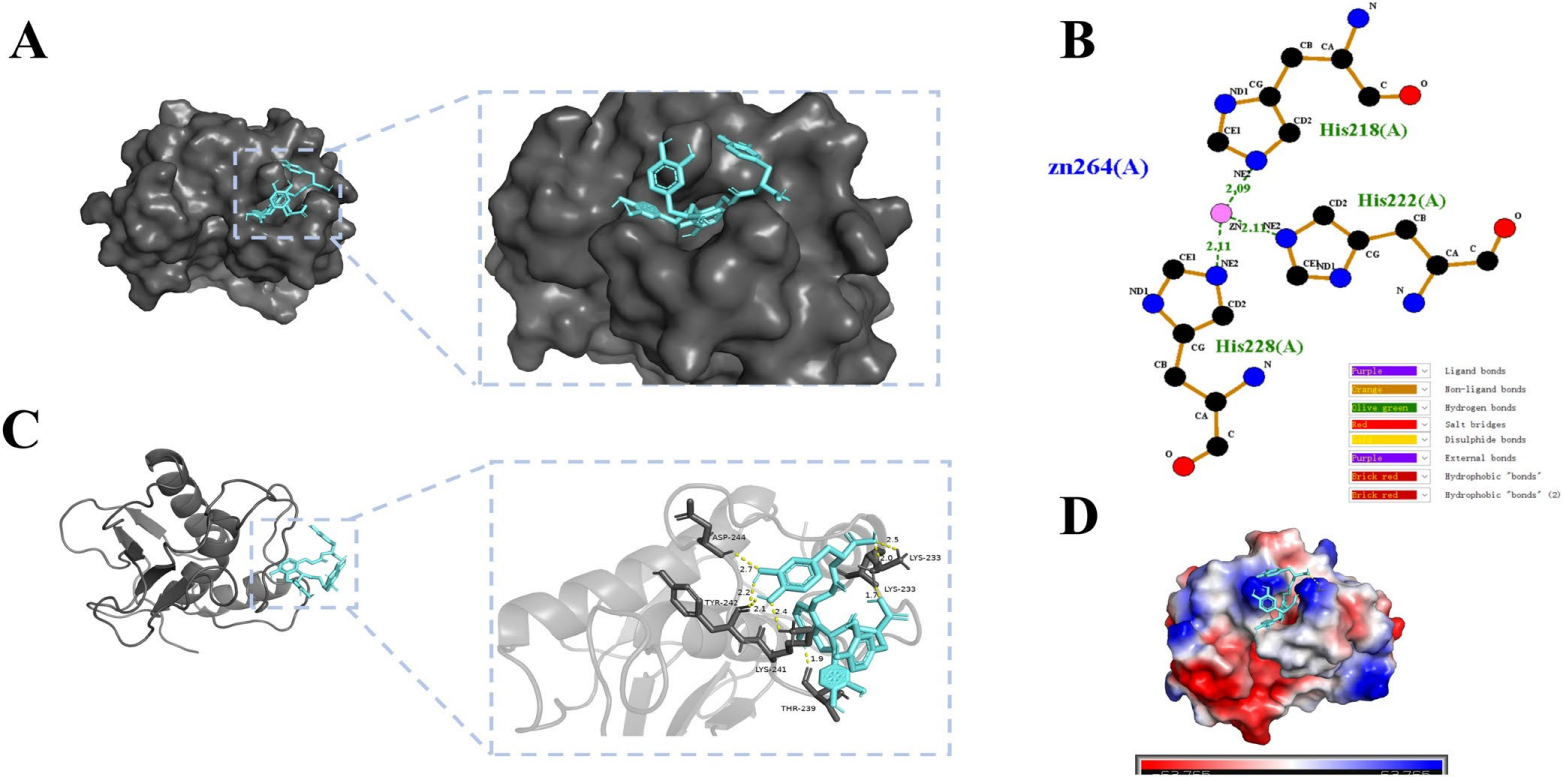
Molecular docking analysis of Sal B with MMP12. (A) Overall binding pose of Sal B (depicted in blue) within the active‐site cavity of the MMP12 protein (rendered as a gray molecular surface). (B) Coordination geometry between the catalytic Zn^2+^ ion (shown as a pink sphere) and the three conserved histidine residues, His218, His222, and His228 (indicated by green dashed lines; bond lengths reported in Å). (C) Hydrogen‐bonding and polar interactions between Sal B and functionally relevant residues of MMP12 (represented by yellow dashed lines; distances labeled in Å). (D) Electrostatic surface potential map of MMP12, highlighting the charge distribution across the Sal B binding region.

### 3.6 Establishment and Validation of MMP12 Overexpression in AC16 Cardiomyocytes

Full‐length validation of the constructed MMP12‐pcDNA3.1(+) recombinant plasmid was performed using Sanger sequencing (Figure S2). The sequence alignment analysis revealed that the inserted MMP12 open reading frame sequence (GenBank accession: NM_002426.2) showed 100% identity with the reference sequence, with no detected base deletions, insertions, or unexpected mutations. The complete reading frame and proper preservation of the restriction enzyme cleavage sites confirmed the successful construction of the recombinant plasmid.

We assessed both transcriptional and translational levels of MMP12 in AC16 cells. RT‐qPCR analysis (Figure 6A) revealed that *MMP12* mRNA expression was markedly upregulated in the MMP12 overexpression group than in the vector group (MMP12 OE, *p* < 0.001). Consistently, western blot analysis (Figure 6B) further confirmed a substantial increase in MMP12 protein expression in the MMP12 overexpression group than in the vector group (*p* < 0.001). These results demonstrate that the MMP12‐pcDNA3.1(+) construct successfully mediates robust overexpression of MMP12 at both the mRNA and protein levels in AC16 cells.

**FIGURE 6:**
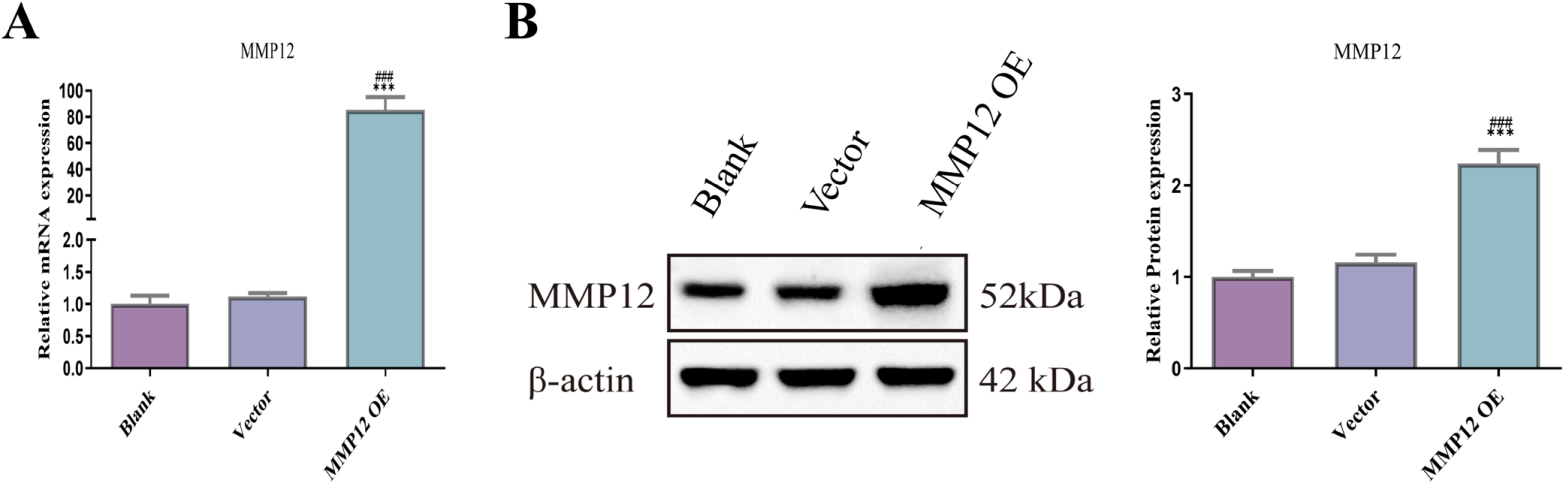
Establishment and validation of MMP12 overexpression in AC16 cardiomyocytes. (A) Reverse transcription quantitative polymerase chain reaction analysis of *MMP12* mRNA expression levels. (B) Quantification of MMP12 protein via western blot. Data are shown as mean ± standard deviation. (***p < 0.001 vs. Blank group; ^###^ *p* < 0.001 vs. Vector group. *n* = 3). MMP12, matrix metalloproteinase 12; OE, overexpression.

### 3.7 MMP12 Overexpression Attenuates the Cytoprotective Effects of Sal B in HG‐Injured Cardiomyocytes

To further clarify the role of MMP12 in the protective effects of Sal B, rescue experiments were performed using MMP12 overexpression. TUNEL staining showed that Sal B reduced HG‐induced cell death, whereas MMP12 overexpression partially reversed this protective effect (Figure 7A). ELISA analysis demonstrated that, compared with the HG group, the group receiving Sal B treatment exhibited significantly decreased levels of IL‐1β, IL‐18, and TNF‐α; however, these inhibitory effects were attenuated after MMP12 overexpression (Figure 7B). Similarly, western blot analysis showed that Sal B treatment reduced the protein levels of NLRP3, ASC, cleaved caspase‐1, and GSDMD‐N in HG‐treated AC16 cells, whereas MMP12 overexpression partially restored the expression of these pyroptosis‐related proteins (Figure 7C). These findings suggest that MMP12 is functionally involved in the protective effects of Sal B against HG‐induced inflammatory injury and pyroptosis‐related signaling in AC16 cardiomyocytes.

**FIGURE 7:**
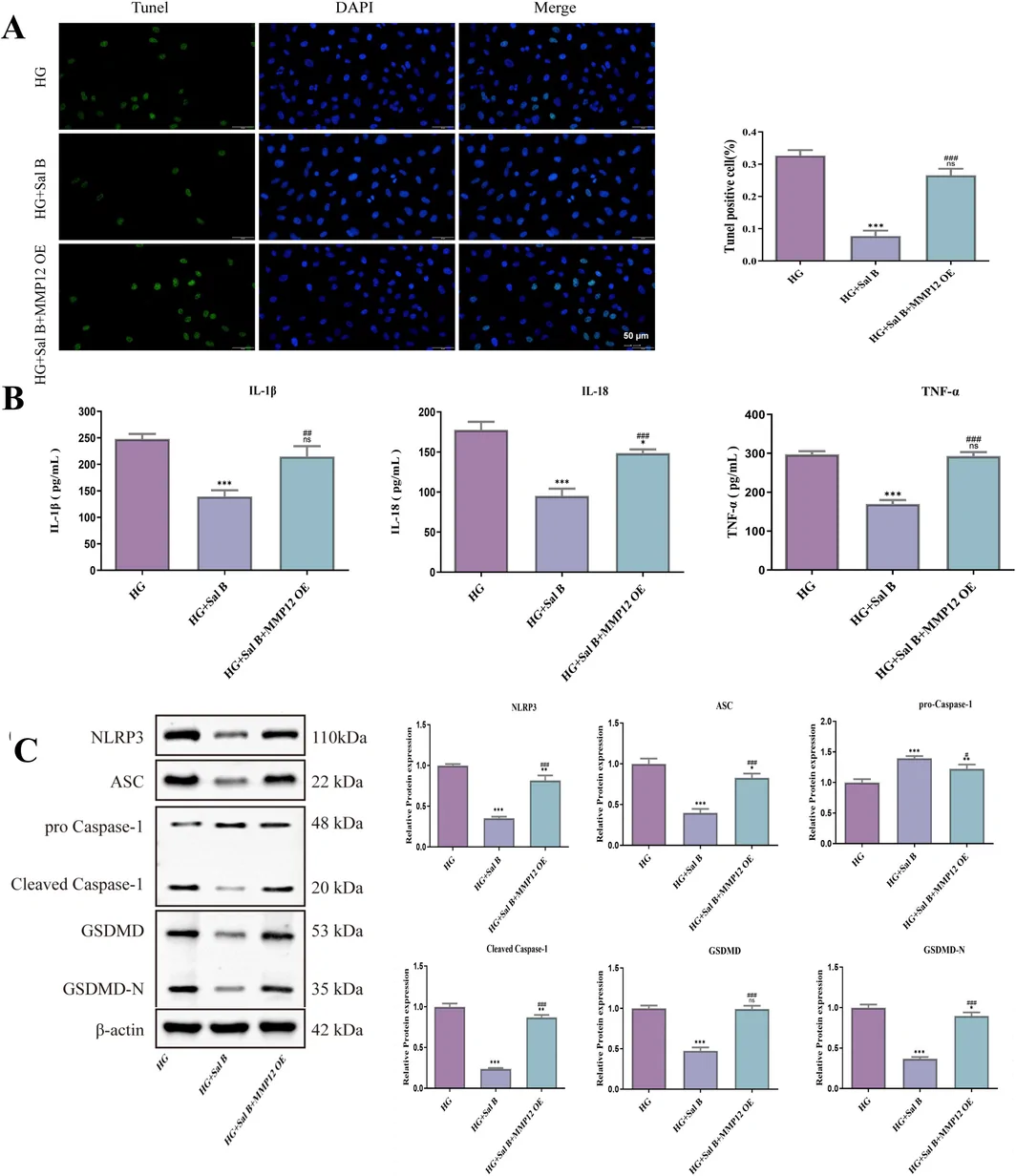
MMP12 overexpression attenuates the cytoprotective effects of Sal B in HG‐injured cardiomyocytes. (A) TUNEL staining analysis quantifying DNA fragmentation as an indicator of cell death in AC16 cardiomyocytes. (B) Levels of IL‐1β, IL‐18, and TNF‐α. (C) Western blot analysis and quantification of protein levels of NLRP3, ASC, pro‐caspase‐1, cleaved‐caspase‐1, GSDMD, and GSDMD‐N. Data are shown as mean ± standard deviation. (There are three groups: HG, HG + Sal B, and HG + Sal B + MMP12 OE. *p < 0.05, **p < 0.01, ***p < 0.001 vs. HG group; ^#^ *p* < 0.05, ^##^ *p* < 0.01, ^###^ *p* < 0.001 vs. HG + Sal B group. *n* = 3). ASC, apoptosis‐associated speck‐like protein containing a CARD; DAPI, 4′,6‐diamidino‐2‐phenylindole; GSDMD, gasdermin D; GSDMD‐N, active N‐terminal fragment of GSDMD; HG, high glucose; IL, interleukin; MMP12, matrix metalloproteinase 12; NLRP3, nucleotide‐binding domain, leucine‐rich–containing family, pyrin domain–containing‐3; ns, not significant; OE, overexpression; Sal B, salvianolic acid B; TNF, tumor necrosis factor.

## 4 Discussion

Our study demonstrates that Sal B alleviates HG‐induced oxidative stress and inflammatory injury in AC16 cardiomyocytes. Mechanistically, Sal B functionally modulates MMP12 to suppress the NLRP3 inflammasome and progression of pyroptosis in AC16 cardiomyocytes. These findings indicate that Sal B functions via the MMP12–NLRP3–pyroptosis axis, providing mechanistic evidence for its regulatory role in MCM‐related cardiomyocyte injury.

In metabolic disorder‐related myocardial injury, the imbalance between ROS generation and clearance in cardiomyocytes leads to excessive ROS accumulation, triggering oxidative stress, inflammation, myocardial fibrosis, and other pathological damage (Yurdagul et al. 2020; Zhu et al. 2021). Sal B can effectively scavenge excess ROS and inhibit the activation of the ROS‐JNK/MAPK signaling pathway, thereby attenuating cardiomyocyte apoptosis and oxidative stress‐mediated inflammatory cascades (Shao et al. 2025; Xu et al. 2023). To explore its mechanism of action, we used an HG‐induced cardiomyocyte injury model to simulate MCM in vitro. Our findings showed that HG exposure altered oxidative stress (reduced SOD activity and increased MDA content) and inflammatory injury (elevated levels of IL‐1β, IL‐18, and TNF‐α), accompanied by excessive ROS accumulation. As TUNEL staining reflects DNA fragmentation and cannot exclusively distinguish pyroptosis from apoptosis, the occurrence of pyroptosis was shown by multiple indicators: activation of the NLRP3 inflammasome (NLRP3 and ASC), cleavage of the pyroptosis executor GSDMD (GSDMD‐N), and increased release of proinflammatory cytokines (IL‐1β and IL‐18). Sal B pretreatment simultaneously ameliorated oxidative stress and inflammation in cardiomyocytes under HG conditions, indicating its dual antioxidant and anti‐inflammatory properties, which may enable it to disrupt the vicious cycle of metabolic myocardial injury. Notably, we observed that Sal B downregulated the mRNA and protein expression of pyroptosis‐related markers (ASC, NLRP3, caspase‐1, and GSDMD), thereby alleviating HG‐induced cardiomyocyte pyroptosis and providing mechanistic insights into the modulation of metabolic cardiomyocyte injury progression by Sal B. However, while we investigated the canonical NLRP3 inflammasome‐dependent pyroptosis pathway, the effects of Sal B on non‐canonical pyroptotic mechanisms, including caspase‐4/5/11‐mediated lipopolysaccharide sensing, or on alternative inflammasome complexes such as absent in melanoma 2 (AIM2) were not assessed (Du et al. 2022). Accumulating evidence indicates that both noncanonical pyroptosis and AIM2 inflammasome activation contribute significantly to the pathogenesis of diabetic cardiomyopathy. Therefore, future studies are needed to systematically investigate whether Sal B exerts broad‐spectrum cardioprotection through coordinated modulation of multiple pyroptotic signaling axes.

To explore the potential molecular mediators of the relevant effects of Sal B against high glucose‐induced cardiomyocyte injury, bioinformatics analysis was performed on sequencing data from tissues of patients with metabolic diseases and tissues from mouse models. The results demonstrated that MMP12 expression was significantly upregulated. However, careful consideration is warranted as the GSE76811 dataset was derived from adipose tissue and aortic samples, not from myocardial tissue. Nevertheless, substantial experimental evidence supports the involvement of both MMP12 and NLRP3 in cardiac pathophysiology. MMP12 has been mechanistically linked to cardiac fibrosis (Zhou et al. 2025), whereas NLRP3 is well‐established as a key mediator of inflammasome activation in multiple cardiovascular inflammatory disorders (Toldo and Abbate 2024). Collectively, these findings provide a biologically plausible basis for postulating an MMP12–NLRP3 signaling axis in the heart; however, direct experimental validation, particularly in cardiomyocytes, cardiac fibroblasts, or intact myocardial tissue, remains essential to confirm its existence and functional relevance. Meanwhile, our study of cardiomyocyte injury models revealed that *MMP12* mRNA was upregulated in HG‐exposed cells, and Sal B pretreatment effectively reduced its expression (Figure S1). Initially studied in the context of chronic obstructive pulmonary disease and atherosclerosis, MMP12 has recently been implicated in cardiac pathophysiology (Lindsey 2018; Shelbaya et al. 2024). For example, MMP12 expression is upregulated in postmyocardial infarction cardiac tissues, primarily derived from infiltrating macrophages, and cardiomyocytes themselves can express MMP12 under stress (Mouton et al. 2018). However, the precise relationship between MMP12 and NLRP3 in cardiomyocytes remains to be defined.

To explore the potential structural basis for the interaction between Sal B and MMP12, we performed a molecular docking simulation. The simulation predicted a favorable binding conformation between Sal B and MMP12 via spatial complementarity between its three‐dimensional conformation and the binding pocket of MMP12 (Paust et al. 2023), indicating that Sal B may modulate proteolytic activity by interacting with the catalytic zinc ion active center (Hu, Wen, et al. 2024) or inducing allosteric conformational changes (He et al. 2023), thereby influencing pyroptotic signaling. Next, we generated an MMP12 overexpression plasmid (Figure S2) and conducted rescue experiments to elucidate the functional relevance of MMP12 in Sal B‐mediated cytoprotection in cardiomyocytes. The results demonstrated that MMP12 overexpression partially abrogated the protective effects of Sal B, indicating that elevated MMP12 levels (Figure S3) mitigate the inhibition of pyroptosis by Sal B and compromise its cytoprotective efficacy. These findings highlight the pivotal role of MMP12 in mediating Sal B's mechanism of action.

In summary, this study confirms that Sal B alleviates HG‐induced cardiomyocyte injury by modulating MMP12‐associated pathways and regulating NLRP3 inflammasome activation. These findings provide mechanistic evidence for the cytoprotective effects of Sal B in metabolic cardiomyocyte injury models, which are mediated at least in part through the MMP12–NLRP3 signaling axis, thereby offering new insights for future translational research in cardiometabolic nutrition.

### 4.1 Nutritional Relevance and Translational Limitations

From a nutritional perspective, the findings of this study expand the mechanistic understanding of plant‐derived polyphenols in regulating cardiomyocyte injury under metabolic stress. Sal B, which is a major bioactive component of the edible medicinal plant *S. miltiorrhiza* , modulates the MMP12–NLRP3 signaling axis to alleviate pyroptotic injury. These in vitro mechanistic findings offer preliminary support to further explore the application of salvianolic acids and related polyphenol‐rich ingredients in functional foods and nutritional intervention strategies targeting cardiometabolic health.

Nevertheless, this study has some translational limitations. The Sal B concentration used in this in vitro study (40 μmol/L) is higher than the physiological blood concentration achievable via ordinary dietary intake, given the low oral bioavailability of polyphenols. Thus, this concentration is only suitable for mechanistic exploration in cellular models and cannot be directly extrapolated to human dietary doses. From the perspective of translational research, two strategies can be adopted to narrow the gap between the in vitro effective concentration and in vivo physiological concentration. First, nano‐delivery systems such as liposomes, polymeric nanoparticles, and phospholipid complexes can significantly improve the oral bioavailability and myocardial tissue enrichment of Sal B, enabling it to reach an effective concentration at the target tissue. Second, the in vivo metabolites of Sal B also possess certain biological activities, and the cumulative effect of prototype drugs and active metabolites may jointly exert cardioprotective effects under physiological conditions. Further studies on bioavailability, in vivo dose‐effect relationships, and active metabolite activities are needed to evaluate the efficacy of Sal B and its derivatives at physiologically relevant concentrations. Additionally, the Sal B–MMP12 interaction was inferred only from molecular docking and target predictions, without direct experimental validation. The rescue assay demonstrated that MMP12 is functionally required, yet it could not discriminate whether Sal B acts through direct enzyme inhibition or indirect suppression of MMP12 expression. Biophysical binding assays (e.g., surface plasmon resonance and isothermal titration calorimetry) and fluorogenic substrate‐based activity tests are required to clarify this regulatory mode. The high discriminatory performance of MMP12 observed in the small discovery cohort may also suffer from overfitting (area under the curve = 1.00); considering the sample size and composition of the discovery cohort, further validation of the result in larger independent populations is necessary. Finally, the 30 mM HG condition employed in this study may elicit nonspecific osmotic effects. Under physiological conditions, human plasma osmolality is tightly regulated within a narrow range of approximately 280–310 mOsm/kg H_2_O. The addition of 24.5 mM glucose (relative to the 5.5 mM normoglycemic control) increases extracellular osmolality, potentially inducing cellular osmotic stress, perturbations in cell volume homeostasis, and osmotic injury. Such effects may independently compromise cardiomyocyte viability and modulate inflammatory responses. Follow‐up studies need to utilize mannitol osmotic control groups to exclude the influence of osmotic pressure and further verify the specific toxic effect of HG metabolism on cardiomyocytes. Future investigations are needed to further characterize the mechanism network of Sal B and explore its application potential in cardiometabolic nutrition research.

## Author Contributions

**Yi Gao:** funding acquisition, investigation, formal analysis, methodology, writing – review and editing, supervision, resources. **Zhi‐Kai Yang:** conceptualization, methodology, formal analysis, writing – original draft. **Longhui Zheng:** formal analysis, methodology. **Shengxian Chen:** formal analysis, methodology. **Pinghua Wu:** formal analysis, methodology. **Xiaotong Ye:** conceptualization, methodology, supervision, writing – review and editing. **Zilong Xie:** investigation, formal analysis, methodology, writing – original draft.

## Funding

This study was supported by the Xiamen Science and Technology Project Fund (3502Z20244ZD1329) and Scientific Research Project of Xiamen ChangGung Hospital (CMRPG1E0944).

## Ethics Statement

This study did not involve human participants or animal experiments conducted by the authors. Publicly available datasets from the GEO database were analyzed, and no identifiable personal information was involved.

## Conflicts of Interest

The authors declare no conflicts of interest.

## Supporting information


**Figure S1:** RT‐qPCR analysis of MMP12 mRNA expression. RT‐qPCR detected a highly significant increase in MMP12 mRNA levels in myocardial cells under 200 mmol/L high‐glucose conditions, which was reduced after Sal B treatment. (**p < 0.01, ***p < 0.001 vs. control group; ^#^ *p* < 0.05 vs. HG group).
**Figure S2:** The MMP12 overexpression plasmid.
**Figure S3:** Western blot analysis and quantification of protein levels of MMP12. (**p < 0.01, ***p < 0.001 vs. HG group; ^###^ *p* < 0.001 vs. HG + Sal B group).

## Data Availability

The data that support the findings of this study are available from the corresponding author upon reasonable request.
