# Supplementary material for: Plant‐Derived Polyphenol Salvianolic Acid B Attenuates Cardiomyocyte Pyroptosis by Modulating the MMP12–NLRP3 Signaling Axis
Source: Food Sci Nutr. 2026 Sep 30;14(10):e72300. doi: 10.1002/fsn3.72300 (PMC13624705; doi:10.1002/fsn3.72300)
Supplement: Supplementary file 1 [file FSN3-14-e72300-s001.docx]

**Supplementary Materials**

**Supplementary Figure 1：**

**Supplementary Figure 1：**RT-qPCR analysis of MMP12 mRNA expression. RT-qPCR detected a highly significant increase in MMP12 mRNA levels in myocardial cells under 200 mmol/L high-glucose conditions, which was reduced after Sal B treatment. (**P < 0.01, ***P < 0.001 vs. control group; ^#^P < 0.05 vs. HG group).

**Supplementary Figure 2：**

**Supplementary Figure 2：**The MMP12 overexpression plasmid.

**Supplementary Figure 3：**

**Supplementary Figure 3：**Western blot analysis and quantification of protein levels of MMP12. (**P < 0.01, ***P < 0.001 vs. HG group; ^###^P < 0.001 vs. HG + Sal B group).
